# Identification of disulfidptosis-related genes and subgroups in Alzheimer’s disease

**DOI:** 10.3389/fnagi.2023.1236490

**Published:** 2023-08-04

**Authors:** Shijia Ma, Dan Wang, Daojun Xie

**Affiliations:** ^1^The First Affiliated Hospital of Anhui University of Chinese Medicine, Hefei, China; ^2^Encephalopathy Center, The First Affiliated Hospital of Anhui University of Chinese Medicine, Hefei, China

**Keywords:** Alzheimer’s disease, disulfidptosis, molecular clusters, machine learning, prediction model

## Abstract

**Background:**

Alzheimer’s disease (AD), a common neurological disorder, has no effective treatment due to its complex pathogenesis. Disulfidptosis, a newly discovered type of cell death, seems to be closely related to the occurrence of various diseases. In this study, through bioinformatics analysis, the expression and function of disulfidptosis-related genes (DRGs) in Alzheimer’s disease were explored.

**Methods:**

Differential analysis was performed on the gene expression matrix of AD, and the intersection of differentially expressed genes and disulfidptosis-related genes in AD was obtained. Hub genes were further screened using multiple machine learning methods, and a predictive model was constructed. Finally, 97 AD samples were divided into two subgroups based on hub genes.

**Results:**

In this study, a total of 22 overlapping genes were identified, and 7 hub genes were further obtained through machine learning, including MYH9, IQGAP1, ACTN4, DSTN, ACTB, MYL6, and GYS1. Furthermore, the diagnostic capability was validated using external datasets and clinical samples. Based on these genes, a predictive model was constructed, with a large area under the curve (AUC = 0.8847), and the AUCs of the two external validation datasets were also higher than 0.7, indicating the high accuracy of the predictive model. Using unsupervised clustering based on hub genes, 97 AD samples were divided into Cluster1 (*n* = 24) and Cluster2 (*n* = 73), with most hub genes expressed at higher levels in Cluster2. Immune infiltration analysis revealed that Cluster2 had a higher level of immune infiltration and immune scores.

**Conclusion:**

A close association between disulfidptosis and Alzheimer’s disease was discovered in this study, and a predictive model was established to assess the risk of disulfidptosis subtype in AD patients. This study provides new perspectives for exploring biomarkers and potential therapeutic targets for Alzheimer’s disease.

## Introduction

Alzheimer’s disease (AD) is a prevalent neurodegenerative disorder characterized by progressive cognitive decline, accompanied by a decline in daily living abilities and psychiatric symptoms, and is the most common cause of dementia (McKhann et al., 2011). The incidence of AD is increasing year by year, with approximately 50 million AD patients worldwide, and epidemiological data analysis predicts that the global incidence of AD will double by 2050 (Scheltens et al., 2021). AD was first discovered and reported by Aloïs Alzheimer in 1907, and over the past century, extensive research has been conducted, but the exact mechanism of AD remains largely unknown (Zhang G. et al., 2021), and there is still no effective treatment for preventing or slowing the progression of AD. Studies have shown that the preclinical latency period of AD can reach 20 years, indicating that we have a long time to intervene in the progression of the disease, making the discovery of AD biomarkers even more important.

Disulfidptosis is a newly discovered cell death mechanism that differs from traditional programmed cell death modes such as apoptosis, necrosis, autophagy, NETosis and pyroptosis (Jorgensen et al., 2016). Liu et al. (2023) found that cells with high expression of SLC7A11 undergo a previously uncharacterized form of cell death, called disulfidptosis, under glucose-deprived conditions due to the abnormal accumulation of disulfide molecules. Excessive accumulation of disulfide molecules induces disulfide stress in actin cytoskeleton proteins, resulting in an increase in disulfide bond levels within actin filaments. This leads to filament contraction and eventual disruption of the cellular skeleton structure, ultimately resulting in cell death. Inhibitors targeting specific cell death pathways have been used to treat various diseases, including neurodegenerative diseases (Deng et al., 2023). The discovery of the novel disulfidptosis mechanism of cell death induced by disulfide bonds in the cellular skeleton provides new potential targets for this form of treatment (Machesky, 2023).

Dendritic spines are excitatory synaptic protrusions located on dendritic shafts and are considered as pathological targets in Alzheimer’s disease (Yu and Lu, 2012). Actin is the major cytoskeletal component of dendritic spines (Landis and Reese, 1983). Mounting evidence suggests that the actin cytoskeleton is crucial for synaptic function and plasticity (Selkoe, 2002). Dysregulation of actin cytoskeletal dynamics has been implicated in the pathological development of Alzheimer’s disease (Pelucchi et al., 2020). Disulfide bond accumulation, which is the mechanism of disulfidptosis, can also lead to actin cytoskeleton damage. Thus suggesting a potential link between disulfidptosis and Alzheimer’s disease, although the specific process of the link requires further analysis and investigation.

In this study, we aimed to explore potential mechanisms underlying AD by analyzing differentially expressed genes between normal and AD samples, utilizing the Gene Expression Omnibus (GEO) database. We performed a cross-referencing analysis between the differential genes and those associated with disulfidptosis, aiming to identify the differentially expressed disulfidptosis-related genes (DRGs). Subsequently, we applied various machine learning algorithms to identify key genes and developed a prediction model. The performance of the prediction model was validated using a nomogram and two external datasets. Finally, based on the expression profiles of seven disulfidptosis-related genes, we classified 97 AD patients into two disulfidptosis-related clusters and further evaluated the differences in immune cells between the two clusters, providing a new perspective for better understanding the potential molecular mechanisms underlying the pathogenesis of AD.

## Materials and methods

### Data acquisition and pre-processing

Three raw datasets (GSE132903, GSE48350, GSE5281, GSE33000, and GSE181279) were obtained from the GEO database using the “GEOquery” R program (Davis and Meltzer, 2007). These datasets contain gene expression data from both Alzheimer’s disease patients and normal groups. The GSE132903 dataset contains 97 AD samples and 98 normal samples, the GSE48350 dataset contains 80 AD samples and 173 control samples, and the GSE5281 dataset contains 87 AD samples and 74 control samples, the GSE33000 dataset contains 310 AD samples and 157 control samples and the GSE181279 dataset contains 3 AD samples and 2 control samples.

### Identification of differentially expressed genes associated with AD and disulfidptosis

Differential gene analysis was performed using the R package “limma” (Ritchie et al., 2015), | log2 fold change (FC)| > 0 and *P* < 0.05 were selected as the threshold for differentially expressed genes (DEGs) between AD and normal samples in the dataset. Differential gene expression data were displayed using volcano plots and heatmaps. Gene ontology (GO) enrichment analysis and Kyoto Encyclopedia of Genes and Genomes (KEGG) pathway analysis were also performed using the “clusterProfiler” package (Yu et al., 2012) in R to further investigate the biological roles of DEGs.

### Evaluating the immune cell infiltration

Single-sample gene set enrichment analysis (ssGSEA) was performed using the R package “GSVA.” Twenty-eight immune gene sets were established, and the degree of immune cell infiltration was calculated for each sample based on the expression matrix of each sample (Hänzelmann et al., 2013). Four other algorithms, including quanTIseq, xCell, MCP-counter and Estimating the Proportion of Immune and Cancer cells (EPIC), were used to verify the stability of the ssGSEA results (Liu et al., 2022; Chen et al., 2023a). These analyses were performed by R package IOBR.

### Construction of predictive model based on machine learning methods

According to the study by Liu et al. (2023) and Zhao et al. (2023a), a total of 26 DRGs were identified. By crossing DEGs with DRGs, differentially expressed DRGs were identified. Three machine learning techniques were then used to further screen the potential gene list for AD diagnosis (Yuan et al., 2023). The least absolute shrinkage and selection operator (LASSO) is a regression method that improves prediction accuracy and selects important feature variables by using regularization techniques (Tibshirani, 1996). Support vector machine (SVM) can perform label prediction on feature vectors by establishing a threshold between the two classes (Noble, 2006). Random forest (RF) is a powerful method for predicting continuous variables and providing stable prediction results (Rigatti, 2017). The intersection genes resulting from LASSO regression, SVM and RF analyses were considered as central hub genes for AD diagnosis (Rajab et al., 2023). Next, a nomogram model for AD cluster occurrence was established using the rms R package (version 6.5.0). The pROC R package was used to perform receiver operating characteristic (ROC) analysis to evaluate the performance of the predictive model in discriminating AD from normal samples. The diagnostic value of the predictive model between AD and normal groups was validated using ROC analysis with two external brain tissue datasets, GSE5281 and GSE48350.

### Validation by real-time PCR and differential expression of external datasets

Total RNA was extracted from the blood samples of AD and healthy controls (HCs) using TRIzol reagent Life Technologies (lot number: 248207), following the manufacturer’s instructions. Subsequently, reverse transcription reactions were performed using RNA 1 μg and RevertAidTM M-MuLV reverse transcriptase (TaKaRa). Real-time PCR was conducted using 2 × SYBR Green qPCR Master Mix (High ROX) Servicebio (lot number: LT202201). The expression data were normalized using the 2-ΔΔCt method with β-actin as the internal reference. The primer sequences used for real-time fluorescence quantitative PCR analysis are provided in Table 1. We also performed differential analysis of the hub genes in GSE181279 and GSE33000. GSE181279 performed single-cell RNA-seq analysis. Quality control, data cleaning, and data analysis were performed using R packages such as “dplyr” and “Seurat.” PCA analysis was conducted on the highly variable genes in the HC and AD groups, resulting in 4 clusters (HCs) and 6 clusters (AD), which were projected onto UMAP plots. The expression distribution of the hub genes in the control and AD groups was explored.

**TABLE 1:** Primer sequences for RT-qPCR.

Gene	Amplicon size (bp)	Forward primer (5′→3′)	Reverse primer (5′→3′)
Hu-β-actin	96	CCCTGGAGAAGAGCTACGAG	GGAAGGAAGGCTGGAAGAGT
Hu-MYH9	150	AAGCTGGTATGGGTGCCTTC	CTTGGGCGGGTTCATCTTCT
Hu-MYL6	196	GCATATCCTGTCGGGGTGAC	GCTGACGGCAAACATCATCC
Hu-DSTN	179	TTGCCAGGACAATCATTAACTGC	AATCCCAGTCCTCTCCTCAGA
Hu-ACTN4	180	GAACCGCTCGAAGTCCACAC	TGTGGCATTCATGTCCTCCC
Hu-GYS1	146	CGAATGGGGCGACAACTACT	TCTGTGCCAGGAACTTGCAG
Hu-IQGAP1	182	TCAGCCATTGTCAGCTCTGT	TCAAAGGCATCAGGAGCAACA

### Subclusters analysis with seven disulfidptosis-related genes

Based on the expression profiles of 7 DRGs, we performed unsupervised hierarchical clustering analysis using ConsensusClusterPlus on 97 AD samples. Gene set variation analysis (GSVA) was conducted to elucidate the functional differences between the disulfidptosis subclusters identified through clustering analysis. The files “c2.cp.kegg.v7.4.symbols” and “h.all.v2023.1.Hs.symbols” were downloaded from the MSigDB online database for GSVA analysis. A heatmap was generated to visualize the distinct activity patterns of the two subclusters. DEGs were identified between the two disulfidptosis-related subclusters. Statistically significant values were considered when | log2 fold change (FC) | > 1 and adj. *p* < 0.05. GO and KEGG enrichment analyses were then conducted using the “clusterProfiler” package to describe their biological functions.

## Results

### Identification of DEGs and functional and pathway enrichment analysis

**FIGURE 1:**
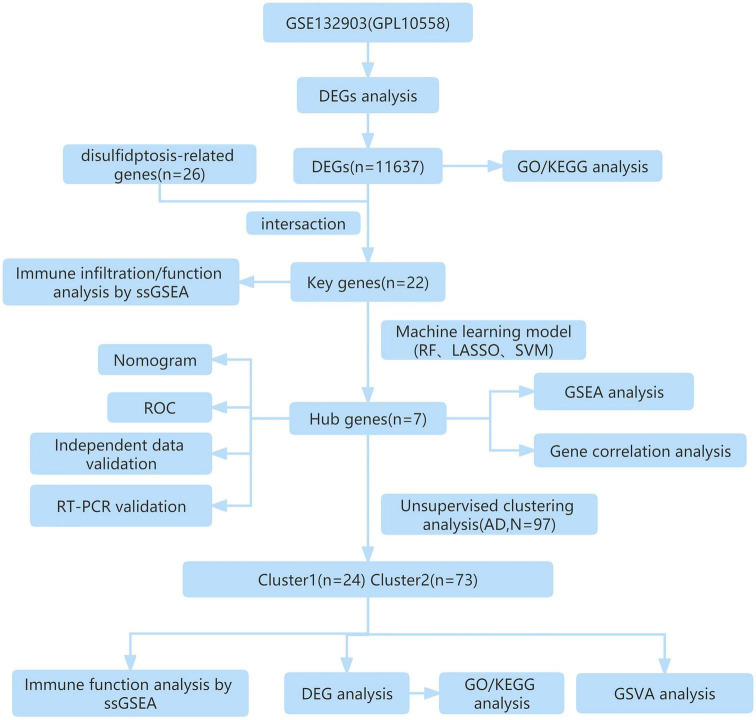
The study flow chart.

**FIGURE 2:**
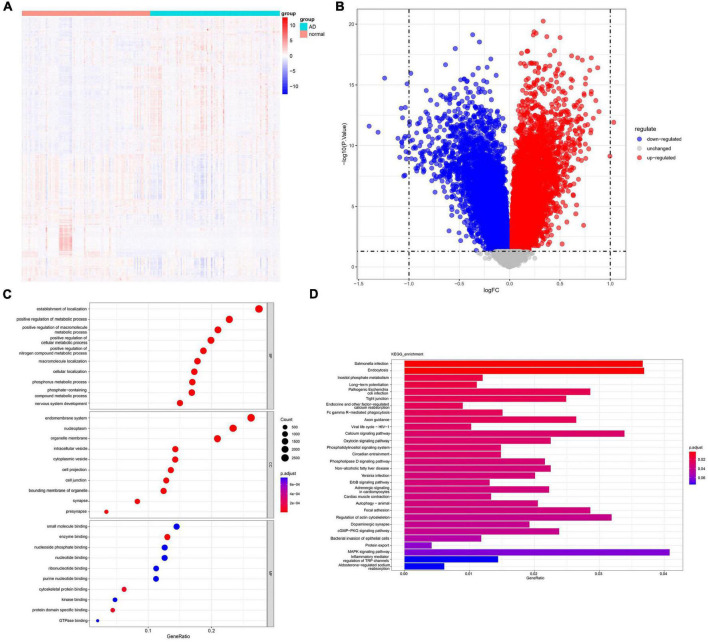
Analysis of differentially expressed genes and enriched pathways in AD. **(A)** An heatmap displays the expression levels of AD-related DEGs. **(B)** A volcano plot highlights the significant DEGs. **(C)** GO analysis shows enriched terms related to AD. **(D)** KEGG pathway analysis reveals enriched pathways in AD. DEGs, differentially expressed genes; GO, gene ontology; BP, biological process; CC, cellular component; MF, molecular function; KEGG, Kyoto Encyclopedia of Genes and Genomes.

The dataset GSE132903 includes 97 AD samples and 98 normal samples. Using the R package “limma,” a total of 11,637 DEGs were identified. Figure 1 illustrates the specific analysis process, while Figures 2A, B display the volcano plot and heatmap of the DEGs, respectively. GO and KEGG enrichment pathway analysis was performed in R to explore the potential role of DEGs. The significant GO-BP (biological process) terms were mainly related to nervous system development, positive regulation of cellular metabolic process, establishment of localization. GO-CC (cellular component) analysis showed that the DEGs were significantly enriched in presynapse, cell junction, cell projection, nucleoplasm. GO-MF (molecular function) enriched pathways were mainly related to cytoskeletal protein binding, small molecule binding, enzyme binding, nucleoside phosphate binding (Figure 2C). The KEGG analysis revealed that these DEGs were enriched in pathways such as salmonella infection, Fc gamma R-mediated phagocytosis, axon guidance, ErbB signaling pathway, autophagy-animal, regulation of actin cytoskeleton and mitogen-activated protein kinase (MAPK) signaling pathway (Figure 2D). These results suggest that these DEGs have important implications for the research and can be further analyzed.

### Evaluation of immune cell infiltration

**FIGURE 3:**
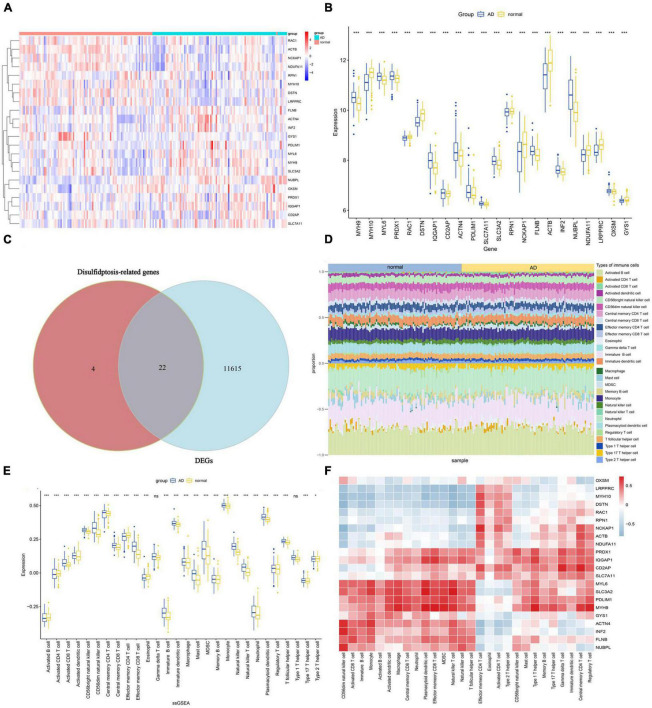
Expression of DRGs and immune cell infiltration in AD. **(A)** Heatmap showing the expression levels of DRGs. **(B)** Boxplot of DRGs expression levels. **(C)** Overlap of genes between DEGs and DRGs. **(D)** The proportions of 28 immune cells infiltrating in AD and normal were compared. **(E)** Boxplots illustrating the differences in immune infiltration between AD and normal. *p < 0.05, ***p < 0.001, ns, no significance. **(F)** Correlation analysis of 22 differentially expressed DRGs and infiltrated immune cells.

The overall expression patterns of 22 DRGs in AD and normal samples are shown in Figures 3A, B. Except for NCKAP1, RAC1, ACTB, NDUFA11, GYS1, DSTN, and MYH10, which are expressed at lower levels in AD, most DRGs are expressed at higher levels in AD. A total of 22 key genes were identified by intersecting the 11,637 DEGs with the 26 DRGs (Figure 3C). To investigate whether there are differences in the immune system between AD and normal groups, an immune infiltration analysis was conducted using the ssGSEA algorithm, which revealed differences in the proportions of 28 infiltrating immune cell types between the AD and normal groups (Figure 3D). The results showed that AD patients had higher levels of activated CD8 T cell, CD56bright natural killer cell, CD56dim natural killer cell, central memory CD8 T cell, effector memory CD8 T cell, immature B cell, immature dendritic cell, mast cell, MDSC, natural killer cell, natural killer T cell and plasmacytoid dendritic cell, while gamma delta T cell and Type 1 T helper cell did not show significant differences (Figure 3E). To gain further insights into the tumor microenvironment (TME) contexture, we employed four additional methodologies, namely xCell, MCPcounter, EPIC, and quanTIseq (Supplementary Figure 1). Furthermore, the correlation analysis results also showed that Immune cells are closely associated with DRGs (Figure 3F). These results indicate that distinct immune cell types display unique infiltrations in AD patients, and DRGs may serve as a crucial factor in regulating the immune infiltration status of AD patients.

### Identification of the disulfidptosis-signature via machine learning

**FIGURE 4:**
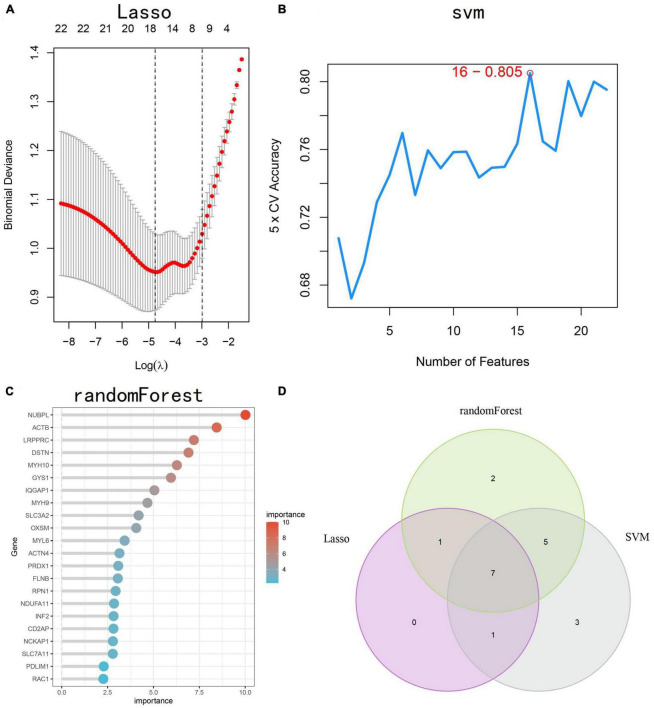
Identification of disulfidptosis-signature using machine learning. **(A–C)** Three different algorithms, LASSO regression, SVM, and RF, were used to construct disulfidptosis-signatures. **(D)** The Venn diagram shows the overlap of candidate genes identified by the three algorithms. LASSO, least absolute shrinkage and selection operator; SVM, support vector machine; RF, random forest.

**FIGURE 5:**
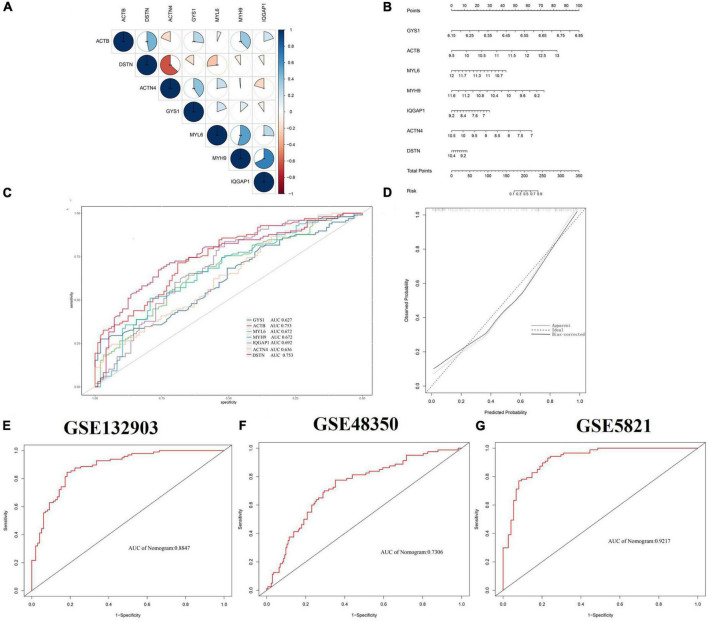
Validation of the diagnostic efficacy based on hub genes. **(A)** Correlation analysis of 7 hub genes. Positive correlations are represented by blue color while negative correlations are represented by red color. The pie chart depicts the correlation coefficients with varying sizes of each slice. **p < 0.05. **(B)** Creating a nomogram to predict the risk of AD using hub genes. **(C)** ROC curve of hub genes in AD. **(D)** Calibration curve to evaluate prediction efficiency of nomogram model. **(E–G)** ROC curves from GSE132903, GSE48350, and GSE5821, respectively. ROC, receiver operating characteristic.

Based on 22 key genes, we used LASSO regression, random forest and SVM algorithms to screen for potential genes and construct a disulfidptosis-signature (Figures 4A–C). Ultimately, we identified eight disulfidptosis-related feature genes as hub genes, including MYL6, MYH9, IQGAP1, ACTN4, GYS1, DSTN, and ACTB (Figure 4D). We conducted gene set enrichment analysis (GSEA) analysis on the seven hub genes, which showed that these genes exhibit enrichment in immune and metabolic related pathways (Supplementary Figure 2). Most of the hub genes were highly correlated with each other and IQGAP1 had a strong synergistic relationship with MYH9 (coefficient = 0.68), while DSTN had a strong antagonistic relationship with ACTN4 (coefficient = 0.63) (Figure 5A). The diagnostic ability of each feature gene in predicting AD was evaluated through ROC curve analysis, and a nomogram model was developed as a predictive tool for AD diagnosis (Figure 5B). The AUC value of the ROC curve for GYS1, ACTB, MYL6, MYH9, IQGAP1, ACTN4 and DSTN were 0.627, 0.753, 0.672, 0.692, 0.637 and 0.753, respectively (Figure 5C). The calibration curve shows that the error between the actual risk and the predicted risk is small, which indicates that the nomogram model has good prediction accuracy (Figure 5D). The AUC value of the ROC curve for nomogram was 0.8847 (Figure 5E). Further validation with external datasets GSE48350 and GSE5281 confirmed the diagnostic value of hub genes, with an AUC of 0.7306 for GSE48350 and 0.9217 for GSE5281 (Figures 5F, G). These results indicate that the nomogram model have good diagnostic value, and we can infer that these genes can accurately distinguish the AD group from the normal group.

### Validation of hub genes expression through real-time PCR and differential analysis with external datasets

**FIGURE 6:**
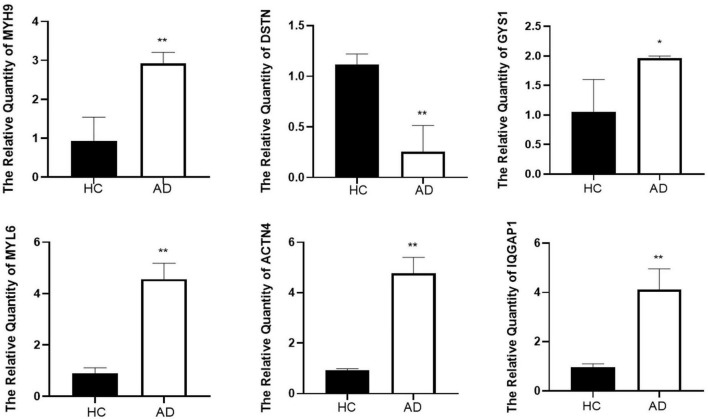
The result of quantitative real-time reverse transcription-polymerase chain reaction (qRT-PCR) illustrated the expression levels of hub genes in patients with AD (*n* = 3) and HC (*n* = 3). *p < 0.05, **p < 0.01.

**FIGURE 7:**
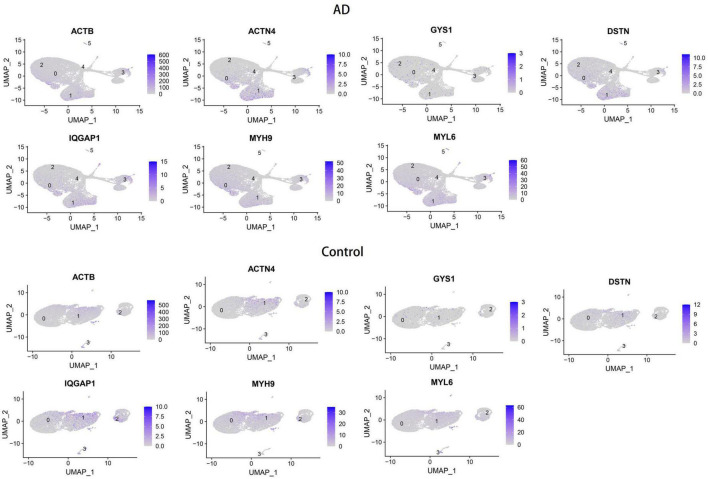
Verification of hub genes expression on single-cell RNA-seq analysis.

To validate the reliability of the hub genes, we collected blood samples from individuals with AD (*n* = 3) and healthy controls (HCs) (*n* = 3) for quantitative real-time reverse transcription-polymerase chain reaction (qRT-PCR). The results showed significantly elevated expression levels of MYH9, MYL6, ACTN4, IQGAP1, and GYS1 in AD patients compared to HCs, while the expression of DSTN was significantly decreased (Figure 6). Furthermore, the expression levels of the seven hub genes were validated in the GSE181279 and GSE33000 datasets. For GSE181279, we performed single-cell RNA sequencing (scRNA-seq) analysis, and quality control, data cleaning, and PCA analysis were conducted as shown in Supplementary Figure 3. The expression and distribution of hub genes are illustrated in Figure 7. The differential analysis results for GSE33000 can be found in Supplementary Table 1. Except for ACTB expression, the results from these two datasets were largely consistent, we considered the discrepancies of ACTB results to the heterogeneity of single-cell sequencing data, as well as differences in experimental methods, technology platforms, data processing, and analysis methods. These results suggest that these genes may have the potential to serve as biomarkers for AD diagnosis.

### Consensus clustering analysis of disulfidptosis gene clusters

**FIGURE 8:**
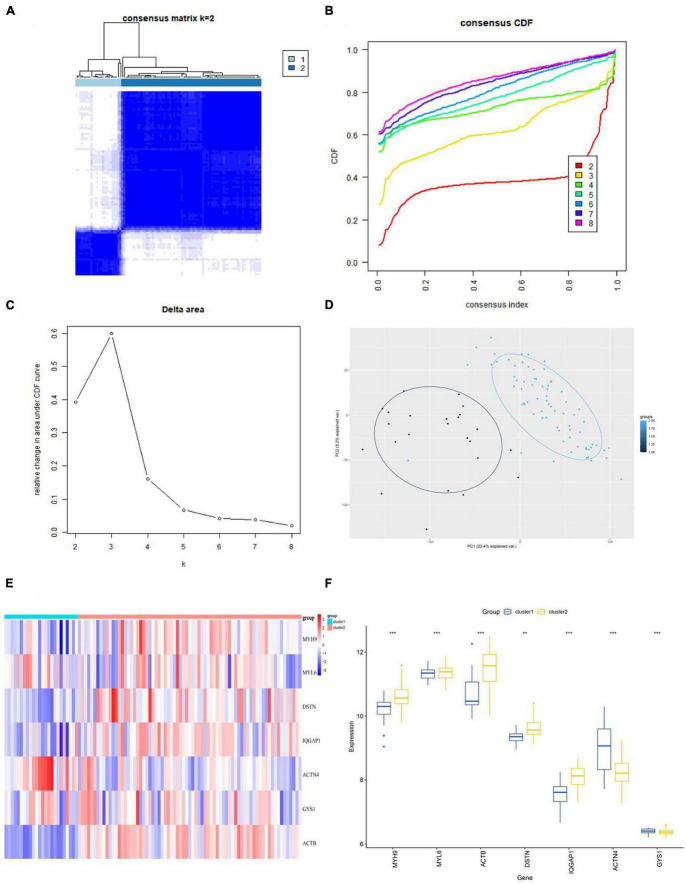
Identification of disulfidptosis subtypes in AD. **(A)** Consensus matrix displaying consensus values when *k* = 2. **(B,C)** CDF and CDF delta area curves were plotted to assess the quality of consensus clustering. **(D)** PCA diagram s depicting the distribution of different subclusters. **(E,F)** Differential expression of DRGs between subtypes is shown in the heatmap **(E)** and boxplot **(F)**. **p < 0.01; ***p < 0.001; ns, no significance. CDF, cumulative distribution function; PCA, principal component analysis.

We performed consensus clustering analysis using the “Consensus Cluster Plus” package in R. Based on the expression profiles of the 7 hub genes, we grouped 97 AD samples. The stability of the clustering was found to be highest when *k* = 2, as demonstrated (Figure 8A), and the CDF curve showed fluctuations within the smallest consensus index range of 0.2 to 0.6 (Figures 8B, C). The same result was achieved from Nbclust testing (Supplementary Figure 4). We ultimately divided the 97 AD patients into two groups, namely Cluster1 (*n* = 24) and Cluster2 (*n* = 73). As shown in the PCA plot, the gene expression patterns between the clusters were distinct (Figure 8D). The expression levels of DRGs in the two subtypes were visualized by heatmap and boxplot (Figures 8E, F). With the exception of ACTN4 and GYS1, most DRGs had higher expression levels in Cluster2, including MYH9, MYL6, ACTB, DSTN, and IQGAP1.

### GSVA of biological pathways between subclusters of disulfidptosis

**FIGURE 9:**
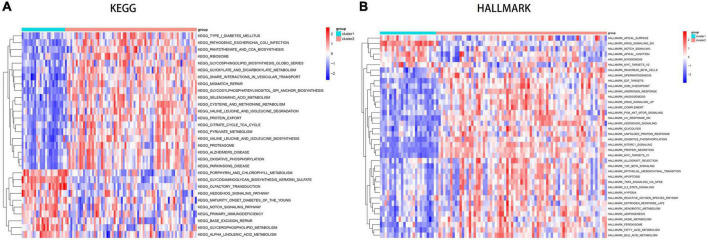
Analysis of key pathways between disulfidptosis subtypes using GSVA. **(A)** Pathway enrichment analysis based on the KEGG database. **(B)** Pathway enrichment analysis based on the Hallmark database.

Using GSVA analysis, we identified several enriched pathways that showed differential expression between the two subtypes, as visualized in a heat map. Based on KEGG pathways, Cluster1 showed higher expression levels in olfactory transduction, Notch signaling pathway and hedgehog signaling pathway, while Cluster2 showed higher activity in oxidative phosphorylation, valine leucine and isoleucine biosynthesis, cysteine and methionine metabolism, mismatch repair and Alzheimer’s disease (Figure 9A). Cluster2 shows elevated expression in several metabolic pathways (including glucose metabolism, lipid metabolism, and amino acid metabolism), as well as disease pathways such as diabetes, Alzheimer’s disease, and Parkinson’s disease. Compared to Cluster1, Cluster2 showed higher Hallmark activity in PI3K AKT MTOR SIGNALING, MTORC1 SIGNALING, TGF BETA SIGNALING, APOPTOSIS, and IL2 STAT5 SIGNALING pathways (Figure 9B).

### Functional distinctions between the two disulfidptosis subclusters

**FIGURE 10:**
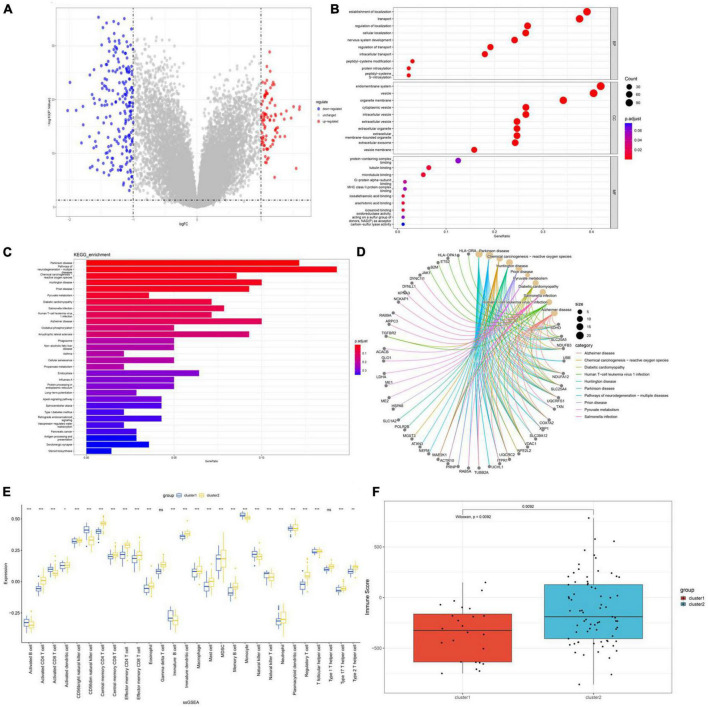
Functional enrichment analysis and infiltration of immune cells in disulfidptosis subtypes. **(A)** The volcano plot of DEGs. **(B)** GO analysis shows enriched terms **(C,D)** Enriched pathways based on KEGG analysis. **(E)** Correlation matrix of all 28 immune cell subtype compositions. **(F)** Boxplots depicting the immune score differences between the two disulfidptosis subtypes. *p < 0.05; **p < 0.01; ***p < 0.001.

To gain further insight into the functional differences between the two subclusters, differential expression analysis was performed and a total of 298 DEGs were identified, including 90 upregulated and 208 downregulated genes. The distribution of these DEGs is shown in the volcano plot (Figure 10A). GO and KEGG enrichment analysis was then conducted on the 298 DEGs to gain a better understanding of potential molecular processes and functions. GO analysis: (BP) shows gene enrichment in protein S-nitrosocysteine, regulation of transport, intracellular transport, nervous system development and intracellular localization; (CC) shows gene clustering in vesicle membrane, extracellular exosome, extracellular membrane-bounded organelle, extracellular vesicle and organelle membrane; (MF) shows cell enrichment in MHC class II protein complex binding, microtubule binding and protein-containing complex binding (Figure 10B). We performed KEGG enrichment analysis and found that these genes were mainly enriched in pathways such as Pathways of neurodegeneration−multiple diseases, Long-term potentiation, apelin signaling pathway and oxidative phosphorylation (Figures 10C, D). Subsequently, we conducted immune infiltration analysis on the two subgroups, and the results showed that the immune microenvironment between Cluster1 and Cluster2 had changed. Activated B cells, activated CD8 T cells, CD56dim natural killer cells, immature B cells, monocytes, natural killer cell and natural killer T cell were expressed at higher levels in Cluster1, while activated CD4 T cells, central memory CD4 T cells, effector memory CD4 T cells, eosinophil, immature dendritic cells, mast cell, memory B cells, neutrophil, regulatory T cells, type 1 T helper cells, type 17 T helper cells and type 2 T helper cells were expressed at higher levels in Cluster2 (Figure 10E). Moreover, the immune score of Cluster2 was relatively higher, indicating that Cluster2 may have a more significant level of immune infiltration (Figure 10F).

## Discussion

As the most common neurodegenerative disease worldwide, Alzheimer’s disease has been extensively studied, and some progress has been made (Zhao et al., 2023b). However, due to the lack of sufficient neurobiological markers and the heterogeneity of the pathogenesis of AD, the current therapeutic effects are still unsatisfactory (Byun et al., 2015; Cano et al., 2021). Therefore, it is crucial to identify more effective diagnostic markers to guide individualized treatment for AD. Disulfidptosis, a recently reported novel form of cell death, is mainly caused by the accumulation of disulfide bonds, leading to cytoskeleton collapse and subsequent cell death, which is closely related to disease progression (Chen et al., 2023b). However, the specific mechanism of disulfidptosis and its regulatory role in various diseases, as well as potential pathways, have not been further studied. Here, we attempted to elucidate the role of disulfidptosis-related genes in AD, linking disulfidptosis to the pathogenesis of AD, and identifying potential key genes through bioinformatics analysis to explore potential therapeutic targets.

In this study, we used the GEO database to investigate the gene expression levels of normal and AD patients and ultimately identified 11,637 DEGs. GO and KEGG enrichment analyses indicated that cells were enriched in positive regulation of metabolic process, cytoskeletal protein binding, MAPK signaling pathway, regulation of actin cytoskeleton and ErbB signaling pathway. Consistent with previous research conclusions that AD patients exhibit metabolic dysfunction in both the central and peripheral nervous system, as well as damage to the actin cytoskeleton in muscle cells leading to synaptic dysfunction and the MAPK and ErbB pathways have been identified as potential therapeutic targets for AD (Lee and Kim, 2017; Clarke et al., 2018; Pelucchi et al., 2020; Zhang H. et al., 2021).

Subsequently, we comprehensively evaluated the expression of DRGs in the brain tissues of both normal and AD individuals. Compared to the normal population, AD patients exhibited more abnormal expression of DRGs, indicating the important role of DRGs in the development of AD. Meanwhile, there were significant changes in the proportion of immune cells between normal and AD patients. Previous studies have shown that the immune system is closely related to AD (Mietelska-Porowska and Wojda, 2017), and our study showed that CD8 T cells, B cells, dendritic cells, mast cells, MDSCs, natural killer cells, and natural killer T cells were more highly infiltrated in AD patients, consistent with previous research (Salminen et al., 2018; Dai and Shen, 2021; Harcha et al., 2021).

In addition, we also found a correlation between these immune cells and DRGs. Furthermore, using three machine learning classifiers, we identified seven hub genes (MYL6, MYH9, IQGAP1, ACTN4, GYS1, DSTN, and ACTB) that are associated with AD. The diagnostic capability was validated using external datasets and clinical samples. Myosin II is an actin-binding protein composed of MHC (myosin heavy chain) IIs, RLCs (regulatory light chains) and ELCs (essential light chains) (Vierthaler et al., 2022). MYL6 is one of the ELCs, while MYH9 and MYH10 are part of the MHC family and encode non-muscle myosin II (NMM-II) which combines with the F-actin network to form the actomyosin cytoskeleton (Pecci et al., 2018; Acebedo et al., 2019); IQGAP1, a key regulatory factor for dendritic spine density, plays an important role in tissue cytoskeleton, microtubule network and cell adhesion by directly binding to actin and promoting actin filament crosslinking, and it also has a specific role in cognitive processes (Fukata et al., 1997; Briggs and Sacks, 2003; Gao et al., 2011; White et al., 2012); ACTN4, an important member of the actin-binding protein family, plays a crucial role in maintaining cell skeleton integrity, controlling cell movement, regulating mRNA metabolism and signal transduction (Khotin et al., 2010; Hsu and Kao, 2013; de Ribeiro et al., 2014); DSTN, a member of the actin depolymerizing factor (ADF)/cofilin family, plays a crucial role in regulating cell skeleton remodeling and actin filament turnover and has been linked to neurological damage (Zhang et al., 2020). DSTN protein has been proposed as a potential biomarker and regulatory protein for AD protection with Rb1 pretreatment (Hwang et al., 2016); Glycogen Synthase (GYS), belonging to the GT3 superfamily, is classified as a retaining glycosyltransferase (GT). Its function involves catalyzing the sequential addition of α-1, 4-linked glucose residues to the non-reducing end of a growing polysaccharide chain. GYS1, belonging to the GYS family, exhibits expression in multiple tissues, including muscle and brain (Inoue et al., 1988; McCorvie et al., 2022); beta-actin (ACTB) is a highly conserved cytoskeletal protein that plays a crucial role in cell growth and cell migration (Gu et al., 2021). ACTB is involved in various cellular processes, including cell motility, intracellular transport, and signal transduction. Its expression and function are essential for the proper functioning and dynamics of cells. The correlation analysis showed significant synergistic or antagonistic effects between these hub genes. Building a diagnostic model using these 7 hub genes may be useful in guiding the diagnosis of AD in clinical practice.

We used unsupervised clustering methods based on the expression of 7 features of double disulfidptosis-regulating factors to estimate molecular patterns in AD brain tissue and ultimately identified two distinct molecular subtypes. Immune infiltration analysis showed that Cluster2 had higher immune scores and relatively higher levels of immune infiltration. Moreover, most DRGs were expressed at higher levels in Cluster2. The KEGG analysis results highlight the close relationship between the DEGs of Cluster2 and Cluster1 and various processes, including metabolism, signal transduction, diseases, and neurodegeneration. The activation of the PI3K/Akt pathway has been shown to have beneficial effects on neurons and neural stem cells (Kitagishi et al., 2014; Razani et al., 2021), while dysfunction of the TGF-β/TβRII signaling axis in the AD brain may accelerate Aβ deposition and neurodegeneration (Das and Golde, 2006; Dammer et al., 2022). The GSVA analysis indicated an upregulation of Cluster2 in TGF BETA SIGNALING and PI3K AKT MTOR SIGNALING.

This study has some limitations that need to be emphasized. Firstly, as our current research is based on comprehensive bioinformatics analysis and RT-PCR validation. It lacks more experimental and clinical trial validation, our results need further confirmation. Additionally, it should be noted that the sample size of this study is relatively small, and larger studies are needed to verify the reliability of the results. Furthermore, this study only involved one group of AD patients and controls, without considering the potential effects of different factors such as age, sex, and race on the results. Moreover, more detailed clinical information is needed to verify the predictive performance of the model, and more external validation cohorts are needed to ensure the stability of the diagnostic model. Finally, more AD samples are needed to clarify the accuracy of the clustering related to disulfidptosis.

## Conclusion

In summary, our study has revealed the correlation between the genes related to disulfidptosis and infiltrating immune cells, and identified 7 feature genes associated with disulfidptosis, which accurately assess the AD subtypes and diagnose AD patients. Moreover, we have elucidated significant immune heterogeneity among different disulfidptosis subtypes of AD patients. Our study has, for the first time, revealed the involvement of disulfidptosis in the development of AD, providing new insights into the potential pathogenic processes and therapeutic strategies for AD.

## Data availability statement

Publicly available datasets were analyzed in this study. This data can be found here: https://www.ncbi.nlm.nih.gov/geo/query/acc.cgi?acc=GSE33000, https://www.ncbi.nlm.nih.gov/geo/query/acc.cgi?acc=GSE181279. The accession numbers: GSE132903, GSE48350, GSE5821, GSE33000, and GSE181279.

## Ethics statement

The studies involving human participants were reviewed and approved by the Medical Ethics Committee is affiliated to The First Affiliated Hospital of Anhui University of Chinese Medicine. The patients/participants provided their written informed consent to participate in this study.

## Author contributions

SM designed the study, collected the original data, finished the analysis, and drafted the initial manuscript. DW helped revise the manuscript. DX provided the funding and supervised the study. All authors read and approved the final manuscript.
