# Supplementary material for: Identification of disulfidptosis-related genes and subgroups in Alzheimer’s disease
Source: Front Aging Neurosci. 2023 Aug 4;15:1236490. doi: 10.3389/fnagi.2023.1236490 (PMC10436325; doi:10.3389/fnagi.2023.1236490)
Supplement: Supplementary file 1 [file Data_Sheet_1.docx]

Supplementary Information

- Supplementary Figure 1
- Supplementary Figure 2
- Supplementary Figure 3
- Supplementary Figure 4
- Supplementary Table 1


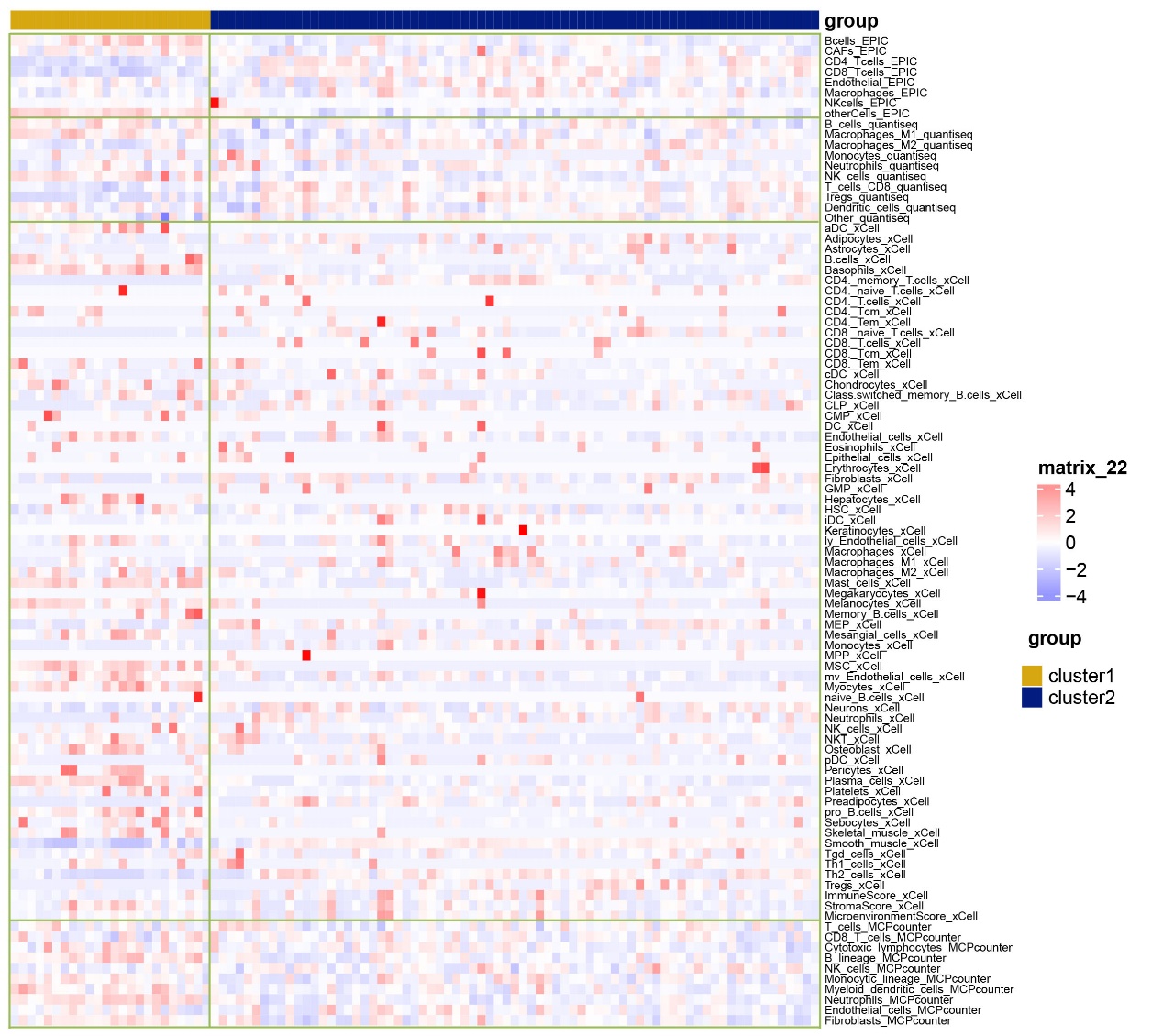


**Supplementary Figure 1**: Four other algorithms including EPIC, quanTIseq, xCell and MCP-counter, further verified the stability and robustness of the ssGSEA results.


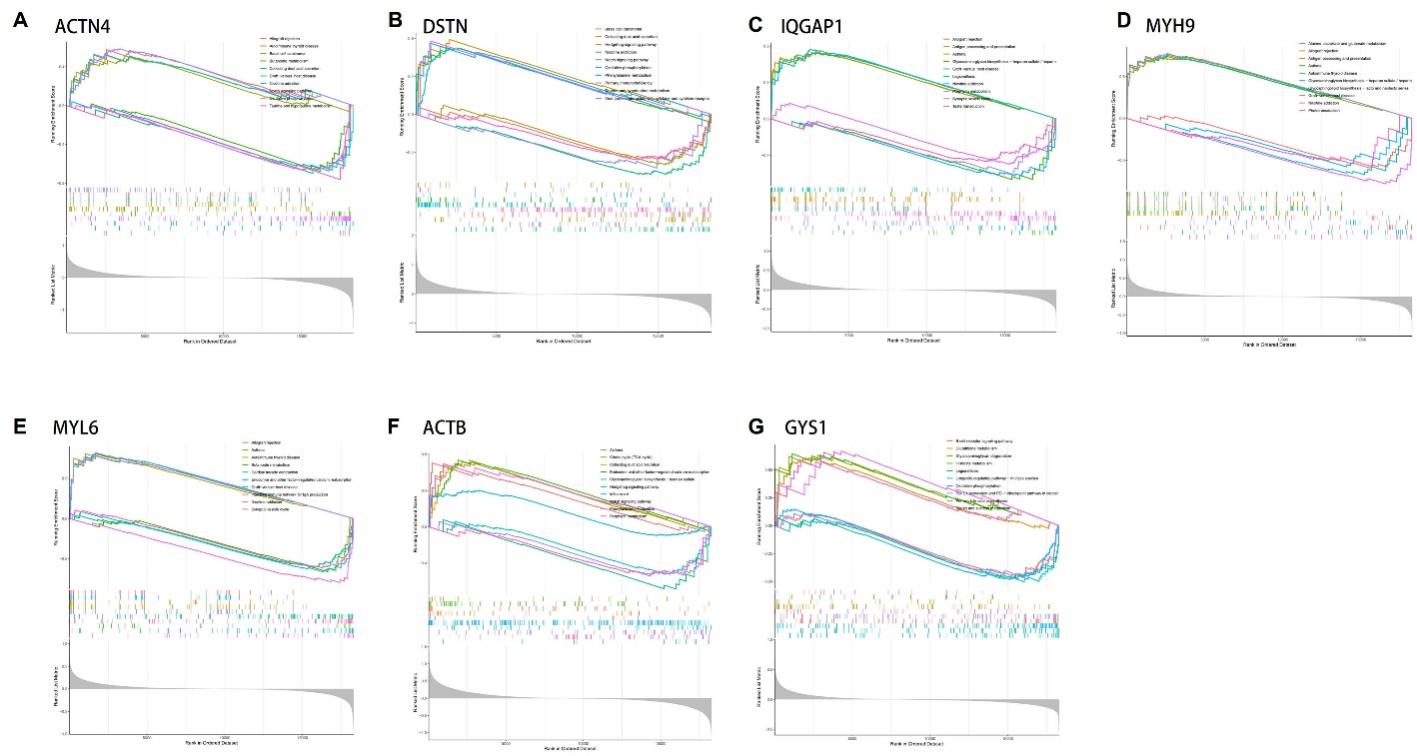


**Supplementary Figure 2:** GSEA investigation of ACTN4, DSTN, IQGAP1, MYH9, MYL6, ACTB and GYS1 (A–G).


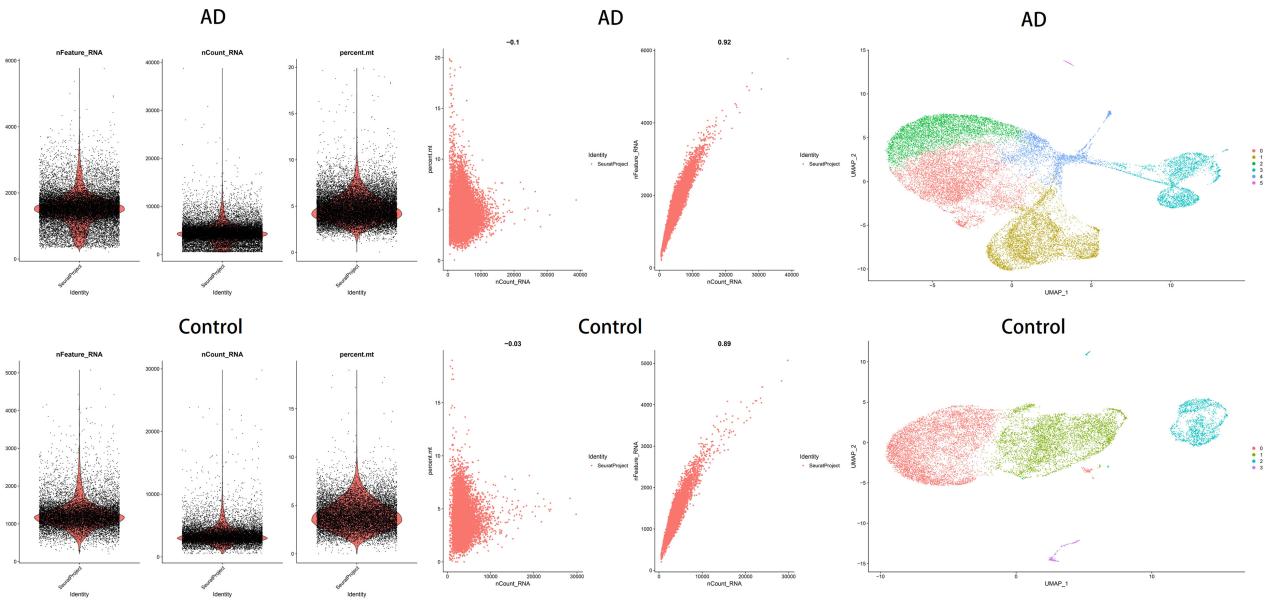


**Supplementary Figure 3:** The quality control, data cleaning, and PCA clustering.


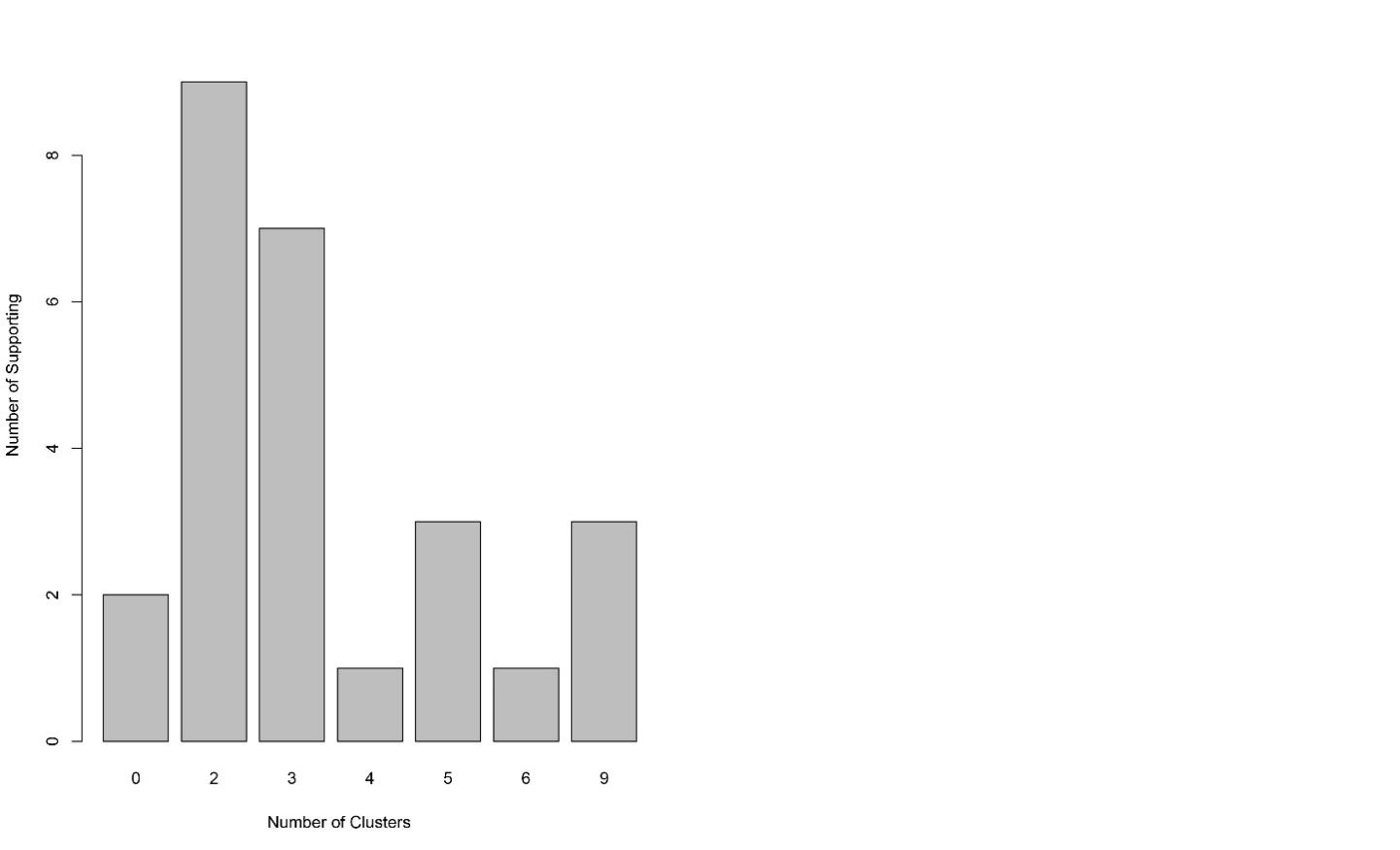


**Supplementary Figure 4:** Recommended number of clusters using 7 hub genes of Nbclust package.


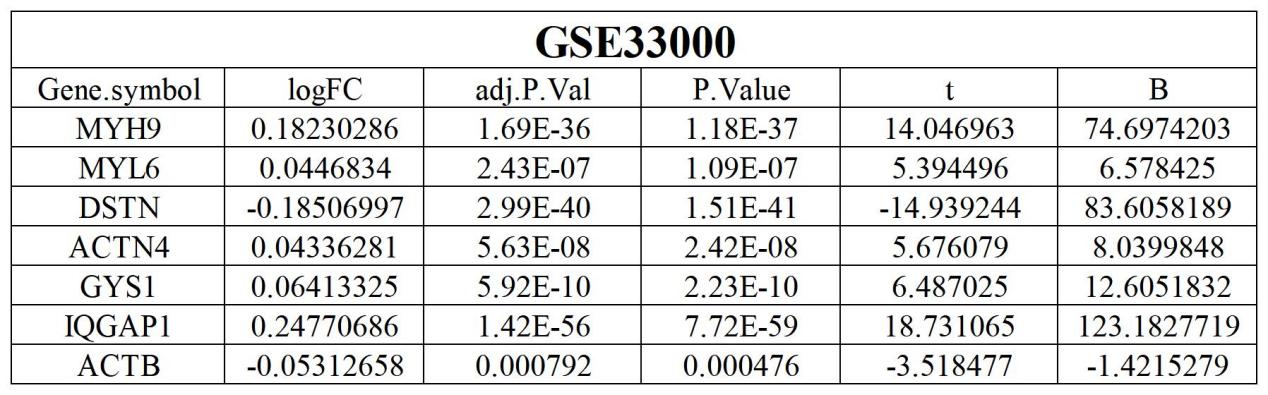


**Supplementary Table 1:** Difference analysis of hub genes in GSE33000.
